# Annotations

**Published:** 1899-05-20

**Authors:** 


### ANNOTATIONS.

### Food Preservatives and the Food and Drugs Amendment Act.

The addition of chemical substances to food materials with a view to preventing their decomposition has become very much more common during recent years. Milk is the chief article of diet which suffers from this form of sophistication, the addition of boracic acid, salicylic acid, bicarbonate of soda, or formalin allowing the dealer to keep the material for quite a lengthened period. In some cases only a small amount of the preservative is added, but frequently it is present in considerable quantities. Thus convictions have been obtained recently against dealers for selling milk adulterated with 32, 59, 68, and 72 grains of boracic acid per gallon. In a case before the Birmingham magistrates it was shown that a sample of butter contained 70 grains of boracic acid per pound, an adulteration equal to 1 per cent. Many samples of butter will be found to contain 20 to 30 grains per pound. The chemical substances we have named above are also very widely used for the preservation of other articles of diet, such as ham, bacon, fish, meat, potted meats, and condensed milk. Boracic and salicylic acid are also added to syrups, lime juices, and other saccharine drinks to prevent fermentation, and are likewise present for a similar reason in many aerated waters, beer, lager beer, coffee extracts, and wines, especially in sweet white wines. Sulphurous acid was formerly used for this purpose, but was less suitable on account of its smell and its effect upon the colour of the red varieties. Preserved, bottled, and canned fruit and vegetables are commonly treated with salicylic acid, an ounce of the drug being used to three pounds of sugar and three gallons of water. Catsup and other condiments also contain this ingredient. Some preservatives are not so innocuous as the above, containing mineral matter not so quickly eliminated from the system. Of these we may mention sodium fluoride, used for admixture with butter, and a meat preservative which analysis showed to contain sulphate of alumina, common salt, nitre, sulphurous and benzoic acids. Under Section VI. of the Food and Drugs Act, 1875, the addition of such preservatives is permitted, provided that they are not injurious to health, and are required for the protection or preparation of the article in a state fit for carriage or consumption. It would be an extremely difficult matter to prove that deleterious effects were experienced from small doses of either salicylic or boracic acid, but when larger quantities are ingested, and the dose is repeated at each meal for a succession of days dyspeptic symptoms may result. With an adulteration of even ·1 per cent. an infant drinking a quart of milk per day might be taking daily doses of 15 to 20 grains of the preservative. In the Birmingham case already quoted a person eating only an ounce of the butter a day would be taking a daily dose of nearly five grains of boracic acid. The symptoms of boracic acid poisoning are vomiting, diarrhœa, hæmorrhage, impaired digestion, and eczematous skin eruptions, with fine scaly exfoliation. Formalin effects more directly the pancreatic digestion of albumins. Where analysis has revealed large quantities of the preservative, convictions for adulteration have been obtained under the Food and Drugs Act, but prosecutions have generally failed when only small quantities have been detected. It is, therefore, a matter for congratulation that the Food and Drugs Act Amendment Bill now before the House of Commons contains a clause providing that any article of food to which a preservative has been added shall bear a label, distinctly and legibly written or printed, stating this fact. In this way the interests of the purchaser will be protected, and the attempt to palm off as fresh articles which are not really so will be frustrated. The proposed clause, however, is hardly stringent enough to guarantee against fraud, and it has been suggested that some words like the following should be added: "The said writing or print to show the nature and proportional quantity of the matter or ingredient added, and the letters on the label intimating the same to be each not less than one-eighth of an inch square." The provision of a Board of Reference to determine the amount and kind, if any, of foreign substances to be allowed for the preservation or flavouring of foods will also be a most valuable addition to our present methods relating to the detection of food adulteration.

### Unqualified Dispensers.

By a question in the House of Commons last Thursday, Major Rasch drew attention afresh to the subject of the employment of unqualified dispensers by medical men. The problem involved is a much more complicated one than some people imagine; and, although not unnaturally, we would gladly see every doctor's surgery provided with a fully-qualified dispenser, anyone who recognises the existing conditions of general practice must know that to expect any such thing would be a purely chimerical idea. There are many who profess to think that it would be to the advantage of the public, and that it would greatly improve the status of medical men if all dispensing were handed over to qualified chemists and druggists, and we can have very little doubt that the repeated references to the subject of "doctors' surgeries" which have recently been made are but part of a policy of pin-pricks by which it is hoped in the end to attain to this consummation. It would, however, be a very serious matter if medical men, especially in the country, were to be driven to give up dispensing. We will not enter upon the question of the greater cost to the public, nor upon that of the quality of the drugs which would be

